# Mechanochemical Activation of DNAzyme by Ultrasound

**DOI:** 10.1002/advs.202306236

**Published:** 2024-02-02

**Authors:** Wolfgang H. Rath, Robert Göstl, Andreas Herrmann

**Affiliations:** ^1^ Institute of Technical and Macromolecular Chemistry RWTH Aachen University Worringerweg 2 52074 Aachen Germany; ^2^ DWI – Leibniz Institute for Interactive Materials Forckenbeckstr. 50 52056 Aachen Germany

**Keywords:** DNAzymes, nucleic acids, polymer mechanochemistry, ultrasound

## Abstract

Controlling the activity of DNAzymes by external triggers is an important task. Here a temporal control over DNAzyme activity through a mechanochemical pathway with the help of ultrasound (US) is demonstrated. The deactivation of the DNAzyme is achieved by hybridization to a complementary strand generated through rolling circle amplification (RCA), an enzymatic polymerization process. Due to the high molar mass of the resulting polynucleic acids, shear force can be applied on the RCA strand through inertial cavitation induced by US. This exerts mechanical force and leads to the cleavage of the base pairing between RCA strand and DNAzyme, resulting in the recovery of DNAzyme activity. This is the first time that this release mechanism is applied for the activation of catalytic nucleic acids, and it has multiple advantages over other stimuli. US has higher penetration depth into tissues compared to light, and it offers a more specific stimulus than heat, which has also limited use in biological systems due to cell damage caused by hyperthermia. This approach is envisioned to improve the control over DNAzyme activity for the development of reliable and specific sensing applications.

## Graphical abstract

The mechanochemical activation of DNAzymes with the help of ultrasound is demonstrated. To deactivate the DNAzyme, it is hybridized to a complementary strand synthesized through rolling circle amplification. DNAzyme activity is then recovered by sonication. The release is mediated by the collapse of cavitation bubbles, which induce shear forces on the RCA backbone.

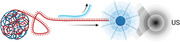

## 1 Introduction

In 1982 the first catalytically active RNA (RNAzyme) was discovered in *Tetrahymena thermophilia*, and over a decade later the first DNAzyme was reported.^[^ ^1^, ^2^ ^]^ They consist, in the case of DNAzymes, of single‐stranded DNA molecules (ssDNAs) that, mostly upon binding to a metal ion cofactor, exhibit catalytic activity for a variety of reactions, including phosphorylation, DNA/RNA ligation, or ester hydrolysis.^[^ ^3^, ^4^ ^]^ The most common subset of DNAzymes are the RNA‐cleaving DNAzymes, which have been utilized in a variety of diagnostic and therapeutic applications.^[^ ^5^ ^]^ They can be used for gene silencing and are therefore a promising tool for cancer gene therapy with high specificity, programmability, and low cytotoxicity.^[^ ^6^ ^]^ For sensing applications, they can be coupled with different readout signals, e.g., fluorescent, colorimetric, or electrochemical.^[^ ^7^ ^]^ DNAzymes exhibit higher chemical and thermal stability than their RNA counterparts enabling their use in biological systems.^[^ ^8^ ^]^

In vivo sensing applications of DNAzymes typically suffer from false positive signaling, because the co‐factors are present in the biological environment, so the DNAzyme activates before reaching its target site. To overcome this problem, a number of caging‐decaging strategies have been developed to enable spatiotemporal control over DNAzyme activity.^[^ ^9^, ^10^ ^]^ These involve the modification of the DNAzyme with a functional group that impedes the catalytic activity and can be cleaved off through an external stimulus. This concept has been realized using light,^[^ ^11^, ^12^ ^]^ heat,^[^ ^13^, ^14^ ^]^ enzymes,^[^ ^15^ ^]^ and reactive oxygen species.^[^ ^16^ ^]^ US is a useful stimulus in biological systems because it offers accurate spatial and temporal resolution and a high penetration depth into tissues compared to other stimuli, e.g., light, enabling non‐invasive treatment.^[^ ^17^ ^]^ Yet, US has rarely been used in the context of DNAzyme activation. Very recently, Lu and coworkers demonstrated the elegant activation of DNAzyme activity through high‐intensity focused US (HIFU).^[^ ^18^ ^]^ There, the DNAzyme is deactivated by hybridization to a protector strand, which is removed by increasing the local temperature above the melting temperature of the double helix with the help of HIFU, thus recovering DNAzyme activity. However, the specificity of heat as stimulus is comparatively low and the induction of hyperthermia is necessary, which can be problematic in biomedical applications leading to cell and tissue damage.^[^ ^19^ ^]^

Conversely, US can be used as a source of mechanical force through acoustic streaming and cavitation.^[^ ^20^ ^]^ The collapse of cavitation bubbles exerts shear force on polymers in solution, resulting in conformational, configurational, and constitutional bond rearrangements along the polymer backbone.^[^ ^21^ ^]^ This enables associated mechanochemical transformations in tailored small molecules at the center of the polymer chain, so‐called mechanophores.^[^ ^22^ ^]^ Polymer mechanochemistry expedites new reaction pathways,^[^ ^23^, ^24^ ^]^ thereby enabling applications of the mechanophore concept as optical force probes^[^ ^25^ ^]^ or in sonopharmacology.^[^ ^26^ ^]^ Recently we have found that the mechanochemistry concept can be expanded to DNA strands with very high molar mass prepared by RCA. During the RCA process, the DNA strands condense into DNA nanoflowers (DNFs) by co‐crystallizing with magnesium pyrophosphate, which is a side product of enzymatic DNA synthesis.^[^ ^27^ ^]^ Due to the dense packing and the absence of nicking sites, DNFs are highly resistant to nuclease degradation.^[^ ^28^, ^29^ ^]^ RCA‐based systems exhibit excellent biostability and biocompatibility and have been used for a variety of bioimaging and therapeutic applications.^[^ ^29^, ^30^ ^]^ We have utilized RCA to design systems for the US triggered release of drugs and enzymes with high specificity.^[^ ^31^, ^32^ ^]^

**Figure 1:**
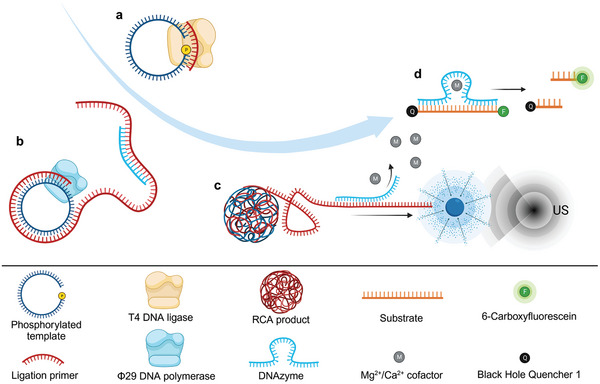
US‐controlled mechanochemical activation of an RNA‐cleaving DNAzyme with the help of RCA products. a) Padlock ligation of RCA template. b) RCA reaction generates a complementary sequence to the DNAzyme and deactivation through hybridization of the DNAzyme to the RCA strand. c) Release of DNAzyme through sonication. d) Cleavage of the substrate labeled with a FRET pair leads to an increase in fluorescence and allows monitoring of DNAzyme activity.

Here we demonstrate the mechanochemical activation of an RNA‐cleaving DNAzyme using US (**Figure** **1**). Therefore, we encode the reverse complementary sequence of the DNAzyme into an RCA product. Deactivation of the DNAzyme is achieved through hybridization to the RCA strand and incorporation into highly condensed DNFs. During ultrasonication, the supramolecular base pair interactions between the RCA product and the DNAzyme are cleaved by mechanical force and the active complex with the cofactor and the substrate is formed. This method offers an alternative pathway to the remote‐controlled activation of DNAzymes by US avoiding non‐specific heating, such as induced by HIFU.

## 2 Results and Discussion

### 2.1 Sequence Design

The DNAzyme consists of two side chains, which bind the substrate by forming a double helix structure, and the active site, which is not involved in Watson‐Crick base pairing. The length and sequence of the side chains can be freely changed to fit the target substrate. The helix formation also works with a large variety of substrates including DNA, RNA, or synthetic nucleotides. The only requirement is a ribonucleotide at the cleavage site. The sequence of the active site is mostly fixed, which was found by mutation studies that show which nucleotides are critical for catalytic activity.^[^ ^33^ ^]^

The previously reported 17E DNAzyme sequence was used as a starting point for our design process.^[^ ^34^ ^]^ The side chains of the DNAzyme were shortened, resulting in a reduction in chain length from 43 to 33. We hypothesized that this would promote the release from the DNFs by reducing the number of base pairs that required cleaving. The sequence of the side chains was altered slightly to avoid the formation of secondary structures, which were calculated using NUPACK. The active site remained unchanged. This sequence was then used for the design of the RCA template. Two spacer regions, a GC‐rich primer binding site and a phosphate group at the 5′‐end were added to complete the template design. The resulting RCA product contained the reverse complementary sequence of the DNAzyme. As a last step, different numbers of mismatches with the DNAzyme were added to the template. We reasoned that this would further promote the release of the DNAzyme and energetically favor binding to the substrate over rebinding to the RCA product.

### 2.2 Optimizing the Padlock Ligation

The first step towards the synthesis of the RCA products was the formation of the circular template. Template and primer strands were mixed in different ratios and a standard annealing protocol was performed (cf. Experimental Section). The optimal ratio between the strands varied, depending on the size of the template and the sequence of the primer. The obtained products were analyzed by agarose gel electrophoresis (Figure S1a, Supporting Information). The main band of the hybridized product showed reduced electrophoretic mobility compared to the linear template, indicating the annealing of the primer (lanes i and ii). With the addition of T4 ligase a further reduction in mobility was observed, suggesting the ligation was successful (lane iii). The closing of the nick reduced the flexibility of the circular construct and therefore its electrophoretic mobility. The presence of higher bands hinted at the formation of larger circular aggregates, such as tetramers or hexamers, and the smearing across the lanes suggested that higher non‐circular aggregates were also formed. The different ligation products were used to perform the RCA reaction, which was analyzed by agarose gel electrophoresis (Figure S1b, Supporting Information). The RCA products had such a high molar mass that they could not enter the pores of the agarose gel. Hence, their exact size and distribution could not be determined. In addition, the formation of a side product with an approximate size of 40 000 nucleotides was observed (lanes iv‐vi). This was explained by the formation of a double‐stranded product, which deactivated the circular template.^[^ ^35^ ^]^ The RCA reaction performed with a template: primer ratio of 2:1 showed the least amount of side product formation, which is why this ratio was chosen for all further experiments.

### 2.3 Controlling DNAzyme Kinetics by RCA

**Figure 2:**
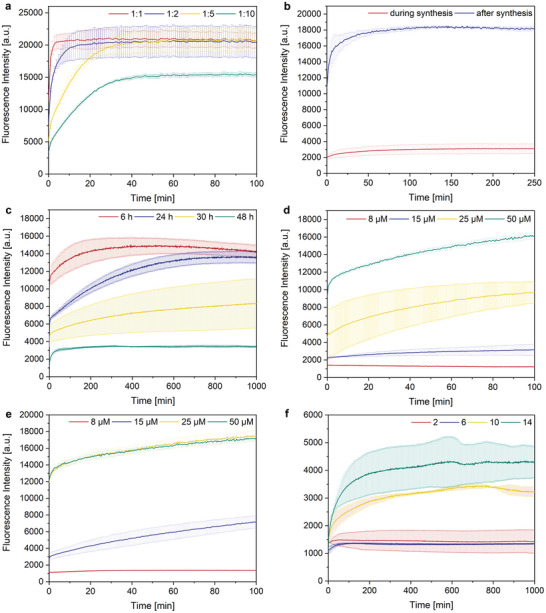
Deactivation of the DNAzyme, 2 µM substrate, all data presented as mean ± SD from the mean, *N* = 3 experiments. a) RNA cleavage kinetics with free DNAzyme. b) Influence of hybridizing DNAzyme to RCA product during/after synthesis, 8 µM DNAzyme. c) Influence of RCA reaction time on DNAzyme deactivation, 8 µM DNAzyme. d) Influence of DNAzyme concentration on deactivation, 2 mismatches. e) Influence of DNAzyme concentration on deactivation, 6 mismatches. f) Influence of mismatches on the DNAzyme deactivation, 8 µM DNAzyme.

To monitor the DNAzymes’ catalytic activity, a substrate labeled with the FRET pair 6‐carboxyfluorescein (6‐FAM) and Black Hole Quencher 1 (BHQ‐1) was used, resulting in the quenching of FAM fluorescence in the unreacted state. Upon substrate cleaving, FAM fluorescence recovered. With addition of increasing amounts of free DNAzyme to a 2 µm solution of substrate, the fluorescence increased concomitantly (**Figure** **2**a). No additional folding protocol was performed for the DNAzyme. At a 1:1 ratio between DNAzyme and substrate, the maximum conversion was reached after ≈ 1 min, and upon full conversion, a 10× increase in fluorescence was observed. After reducing the DNAzyme amount to a 1:2 ratio, full conversion was reached after ≈ 10 min, or in the case of a 1:5 ratio after 30 min. When reducing the ratio to 1:10, a further decrease in activity was observed and the fluorescence plateaued before reaching the maximum, i.e., full conversion. This plateau indicated that the maximum number of catalytic cycles for the 17E DNAzymes lay between 5 and 10.

Next, we investigated the deactivation of the DNAzyme after hybridizing it to the RCA product. A significant difference between hybridizing the DNAzyme to the RCA product post‐synthesis and adding the DNAzyme directly at the start of the RCA reaction was observed (Figure 1b). The RCA reaction was performed with a template including two mismatches (cf. **Table** **1**, template 1) and a reaction time of 36 h. Adding the DNAzyme to the reaction mixture resulted in a significant decrease in catalytic activity, while the post‐synthesis hybridization had almost no effect. This was likely rooted in the co‐crystallization of the enzymatically generated DNA with magnesium pyrophosphate. The adsorption of the RCA chains to the inorganic core led to a dense packing of the DNA, which restricted penetration of the DNFs by the DNAzyme and hence prevented hybridization. Heating to 95 °C for 5 min was not sufficient to denature these structures and hence did not improve the deactivation of the DNAzyme. When adding the DNAzyme directly to the RCA reaction mixture, the hybridization occurred in situ before the formation of the DNFs. The latter approach was used for all further experiments. The loaded DNFs were characterized by scanning electron microscopy (SEM, Figure S2, Supporting Information). Particles with an average diameter of 4.72 ± 2.40 µm and the typical sponge‐like structure were found. We observed the formation of aggregates (Figure S2a, Supporting Information), probably caused by the interaction of DNA strands, that are adsorbed to the particle surface (Figure S2b, Supporting Information).

**Table 1:** DNA sequences.

Name	Sequence (5′→3′)
17E DNAzyme	GCC ATC TTC TCC GAG CCG GTC GAA ATA CTG ACT
17E Substrate	6‐Fam – AGT CAG TAT rAGG AAG ATG GC – BHQ‐1
RCA Template 1	P – CCC TCT CTC CTC AAA AAA AAA AAA TCC ATC TTC TCC GAG CCG GTC GAA ATA CTG AC**A** AAA AAA AAA AAA CTC CTC CTG ACT
RCA Template 2	P – CCC TCT CTC CTC AAA AAA AAA AAA **TG**C ATC TTC TCC GAG CCG GTC GAA ATA CTG AC**A** AAA AAA AAA AAA CTC CTC CTG ACT
RCA Template 3	P – CCC TCT CTC CTC AAA AAA AAA AAA **TG**C ATC TTC TCC GAG CCG GTC GAA ATA CTG A**TA** AAA AAA AAA AAA CTC CTC CTG ACT
RCA Template 4	P – CCC TCT CTC CTC AAA AAA AAA AAA **TGG** ATC TTC TCC GAG CCG GTC GAA ATA CTG A**TA** AAA AAA AAA AAA CTC CTC CTG ACT
RCA Template 5	P – CCC TCT CTC CTC AAA AAA AAA AAA **TGG** ATC TTC TCC GAG CCG GTC GAA ATA CTG **GTA** AAA AAA AAA AAA CTC CTC CTG ACT
RCA Template 6	P – CCC TCT CTC CTC AAA AAA AAA AAA **TGG CG**C TTC TCC GAG CCG GTC GAA ATA C**AC GTA** AAA AAA AAA AAA CTC CTC CTG ACT
RCA Template 7	P – CCC TCT CTC CTC AAA AAA AAA AAA **TGG CGA A**TC TCC GAG CCG GTC GAA AT**G TAC GTA** AAA AAA AAA AAA CTC CTC CTG ACT
RCA Primer	GAG GAG AGA GGG AGT CAG GAG GAG

Loaded DNFs were synthesized with RCA reaction times between 6 and 48 h and the strongest deactivation was achieved after 48 h (Figure 1c). According to the manufacturer of the RCA kit, full conversion should be reached after 10 h, when using 900 pmol of dNTPs and 30 U of Φ29 polymerase. A possible explanation for our observation might be that full deactivation was only reached after complete co‐crystallization of the ssDNA with Mg_2_P_2_O_7_ – a slower process than the DNA synthesis itself. Polymerization kinetics were possibly also negatively affected due to increasing viscosity during RCA, which led to a hydrogel‐like RCA product after 48 h.

Next, the maximum loading amount was determined, at which full deactivation could still be observed (Figure 1d,e). This experiment was performed with templates containing 2 and 6 mismatches (cf. Table 1, templates 1 and 2). As expected, the RCA product containing two mismatches showed better deactivation performance than the product containing six mismatches. At a DNAzyme concentration of 15 µm the RCA product with two mismatches showed almost full deactivation, while with six mismatches significant DNAzyme activity was observed. At 8 µm, both RCA product variants showed full deactivation, hence this concentration was chosen for all further experiments.

Finally, the correlation of the deactivating properties of the RCA product and the amount of mismatches with the DNAzyme were investigated further (Figure 1f). Full deactivation was achieved with 2 and 6 mismatches (cf. Table 1, templates 1 and 2), while a 2–3 fold increase in fluorescence was observed with 10 or 14 mismatches (templates 3 and 4). This was probably caused by spontaneous strand displacement, which became more likely as the length of the toehold on the DNAzyme was increased. All further experiments were performed with two and six mismatches on the RCA product.

### 2.4 Mechanochemical DNAzyme Activation by Ultrasound

**Figure 3:**
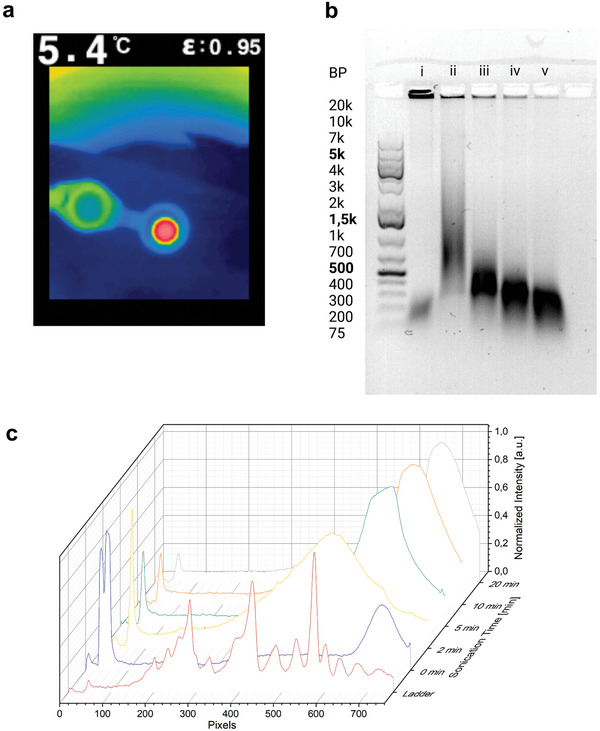
Sonication of DNAzyme‐loaded RCA products. a) Thermal image of reaction mixture immediately after sonication. b) GE of RCA products after sonication; 0.8% agarose; ladder: GeneRuler 1 kb plus i: 0, ii: 2, iii: 5, iv: 10, and v: 20 min sonication time. c) Normalized intensities of GE bands plotted against pixels in *y*‐direction.

After successful deactivation, we investigated the mechanochemical release of the DNAzyme upon US application and the subsequent catalytic conversion of the DNAzyme substrate. The sonication experiments were conducted by adding the loaded RCA product and the substrate to the DNAzyme buffer and then sonicating with a 20 kHz immersion probe sonicator (cf. Experimental Section). The mixture was cooled with ice, and the maximum detected temperature was below 6 °C, measured directly after sonication (**Figure** **3**a).

The size distribution of the RCA products after sonication was analyzed by agarose gel electrophoresis (Figure 3b). We observed a decrease in chain length with increasing sonication time, resulting in a minimal chain length of ≈400 bases. This was caused by covalent bond scission at the DNA backbone through inertial cavitation. After repeated chain fractures, a cut‐off length was reached below which no US responsivity was observed. This observation was consistent with our previous findings.^[^ ^31^ ^]^ To determine the chain length more precisely, the intensities of the bands were quantified using the gel analysis tool in ImageJ, and plotted against the pixels as a measure of relative elution position in the *y*‐direction (Figure 3c). The local intensity maxima of the ladder plot were used for the calculation of a calibration curve to determine the exact DNA chain length depending on the *y*‐position (Figure S3, Supporting Information). The position of the maximum intensities was then used to calculate the exact chain length after sonication.

The DNFs were also characterized with SEM after 20 min sonication (Figure S4, Supporting Information), through which we observed a decrease in average particle size to 2.63 ± 0.70 µm. The particles were compacted, resulting in an oval morphology (Figure S4a, Supporting Information), or completely deformed into irregular shapes (Figure S4c, Supporting Information). Additionally, we observed a distinct lack of aggregates, which were prominent before sonication.

**Figure 4:**
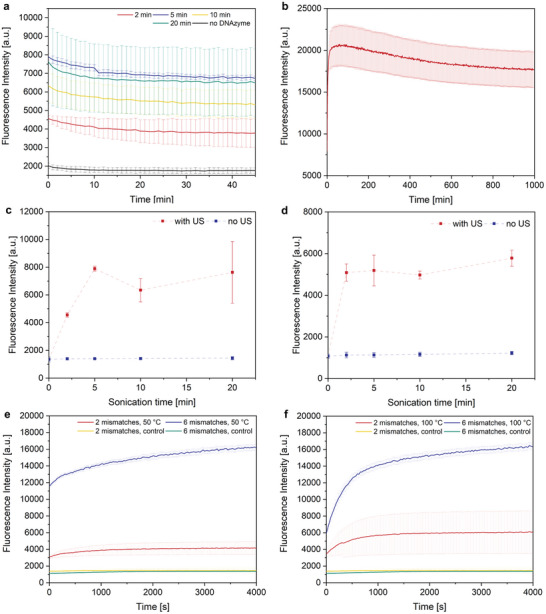
Activation of DNAzyme by US, 2 µM substrate, all data presented as mean ± SD from the mean, *N* = 3 experiments. a) DNAzyme kinetics after varying sonication time, 8 µM DNAzyme. b) Photobleaching behavior of DNAzyme substrate after full conversion. c) Maximum fluorescence observed after sonication, 2 mismatches, 8 µM DNAzyme. d) Maximum fluorescence observed after sonication, 6 mismatches, 8 µM DNAzyme. e) Thermal release of DNAzyme, 50 °C, 8 µM DNAzyme. f) Thermal release of DNAzyme, 100 °C, 8 µM DNAzyme.

Hereafter, the substrate conversion after sonication was assessed through observation of the evolution of the fluorescence intensity after US application (**Figure** **4**a). Surprisingly, we observed the maximum fluorescence directly after sonication, followed by a continuous decrease in the fluorescence signal. The substrate alone showed no increase in fluorescence after sonication, confirming that the active species was rapidly generated in situ, and the maximum conversion was already reached during sonication. We found that the decline in fluorescence intensity over the course of the measurement likely stemmed from photobleaching, which we confirmed in an extended control measurement with the substrate alone (Figure 4b).

We found that the catalytic activity vanished once the sonication was stopped, which we believe was likely caused by the rebinding of the DNAzyme to the fragments of the RCA product (Figure 4c,d). While the DNAzyme could be released again from the rebound state, upon reaching the cut‐off length of the RCA fragments the constructs were no longer responsive toward US and the DNAzyme was permanently deactivated. Even though the binding of the DNAzyme to the substrate was energetically favored because of the mismatches on the RCA product, the concentration of the RCA fragments was considerably higher compared to the substrate, so that rebinding was kinetically favored.

In the case of two mismatches, the maximum intensity was reached after 5 min US, while with six mismatches, the maximum was already reached after 2 min US. A similar behavior was observed with templates containing 3, 4, and 5 mismatches (Figure S5b, Supporting Information), which all reached the maximum intensity after 2 min sonication. The mismatches appeared to accelerate the release process while having no significant influence on the maximum conversion of 30–40% of the full conversion (Figure 1a), where no RCA product was present. This finding was explained by the rebinding mechanism lowering the effective concentration of active species.

To support our rebinding hypothesis, we compared the sonication results to a thermal release process. In contrast to the mechanochemical release mechanism, the RCA products did not fracture during the thermal release and remained in their condensed DNF state, leading to a considerably lower chance of rebinding. We observed a steeper increase in catalytic activity in the case of 6 mismatches compared to 2 mismatches, confirming that the additional mismatches promoted the release by lowering the energy barrier for activation (Figure 4e,f). Moreover, we recorded an overall higher fluorescence intensity after heating in the case of 6 mismatches, with a maximum conversion of ≈85%. This implied that the catalytically active species were still present and supported our hypothesis that the rebinding was considerably less pronounced in the presence of intact DNFs.

Because of the contrasting DNAzyme kinetics after heat‐ and US‐triggered release, we inferred a different activation mechanism between the two methods. Especially the rebinding process hinted at the mechanochemical nature of the US release. If the release was purely based on the melting of the double helix formed between DNAzyme and RCA product, a constant re‐release would be possible, regardless of chain length. The mechanochemical activation on the other hand, required a minimum chain length to exert shear forces on the RCA product. Once the minimum chain length was reached, the DNAzyme was permanently deactivated.

**Figure 5:**
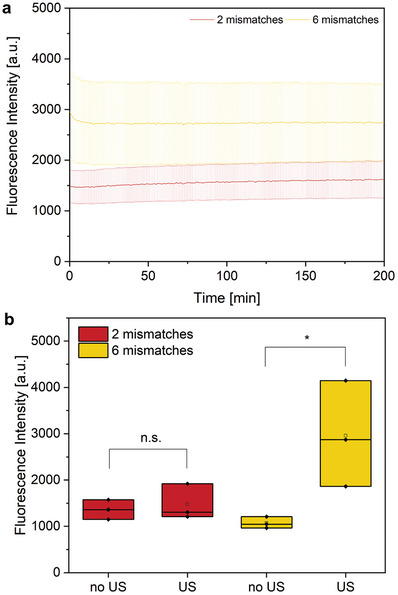
Fluorescence intensity after 10 min HIFU sonication, 2 µM substrate, 8 µM DNAzyme. All data presented as mean ± SD from the mean, *N* = 3 experiments, *p* = 0.047, calculated using an unpaired two‐sample t‐test, ^*^ *p* ≤ 0.05. a) DNAzyme kinetics after HIFU sonication. b) Maximum fluorescence intensities after HIFU sonication.

After demonstrating successful activation of the DNAzyme through 20 kHz US, we explored whether the same is possible with more biocompatible US sources (**Figure** **5**). We exposed our samples to HIFU at 0.66 MHz and a focal pressure of 1940 kPa for 10 min. Similar to the 20 kHz experiments, the maximum fluorescence was achieved immediately after sonication, hinting at a mechanochemical release process. We found the activation efficiency to be heavily dependent on the amount of mismatches of the DNAzyme with the RCA product. In the case of 2 mismatches, we observed no activation, while with 6 mismatches moderate, but statistically significant activation (*p* = 0.047) could be achieved. The chosen US parameters resulted in a mechanical index (MI) of 2.38, indicating the presence of cavitation bubbles in the solution. This MI exceeded the FDA safety guidelines for imaging US (1.9), still we could achieve an ≈89% reduction in MI compared to the 20 kHz US (20.46). The possibility of activation with HIFU and the low cytotoxicity of our system indicated potential for the future use in biological systems.

With this approach, we could expand the scope of DNAzyme activation by external stimuli. Through the use of US, it might be possible to achieve deeper penetration depths than light and DNAzyme activation in non‐transparent media. We could show very reliable deactivation of the DNAzyme through non‐covalent interactions, even at full co‐factor concentration. Release of the DNAzyme was very reliable with 20 kHz US and moderately successful with HIFU. We were able to activate our system with much lower sonication times compared to the HIFU‐mediated release by Lu and coworkers. Additionally, we achieved complete independence of temperature, which could solve problems caused by extended US‐induced hyperthermia in the future.

## 3 Conclusion

DNAzymes are a versatile tool for the catalysis of a variety of reactions and a promising agent for biomedical applications. We successfully established a system to control the activity of a DNAzyme by US relying on the mechanochemical activation from DNF carriers. First, we optimized the deactivation of the DNAzyme through hybridization to the RCA product by investigating the RCA reaction time, mismatches, the addition timing of the DNAzyme, and the loading amount. The deactivation was mediated by hybridization to the RCA strand, as the mismatch studies showed. The condensation process to form DNFs also played a role, as the reaction time studies suggested. In combination with the addition timing, we inferred that hybridization between the DNA strands occurred rapidly, followed by a slow co‐crystallization process with Mg_2_P_2_O_7_ to form DNFs. Upon completion of this process, the binding to the substrate through spontaneous strand displacement was suppressed. In addition, we successfully demonstrated the reactivation of the DNAzyme through ultrasonication with 20 kHz US, and we gained insight into the release and rebinding dynamics during the sonication process. We observed significant substrate conversion after sonication, underlining that the DNAzyme was released and the active species was formed in situ. Conversion stopped after sonication, highlighting that the DNAzyme was deactivated by re‐binding to the RCA product, which fragmented through covalent bond scission at the phosphate backbone. The reaction was then reinitiated through continuous re‐release during sonication until the RCA fragments were no longer US‐responsive and the DNAzyme was sequestered. We did not observe this effect for the concurrent thermal release, where intact DNFs were maintained, suggesting that the fragmentation of the RCA product during sonication caused the re‐binding to be kinetically favored. The deactivation through the RCA fragments underlined the mechanochemical nature of the release process, since the thermal release would be possible with shorter fragments. This constitutes an unprecedented pathway to exert temporal control over the activity of DNAzymes. Moreover, we demonstrated DNAzyme release through HIFU application at a frequency of 0.66 MHz with an MI of 2.38. At this MI inertial cavitation is probable, which we believe to be crucial for the mechanochemical release mechanism since higher frequencies and lower MIs did not lead to a successful DNAzyme activation.^[^ ^36^, ^37^ ^]^ Although this was a significant improvement compared to 20 kHz US for potential biocompatibility, the inherent reliance on inertial cavitation will prohibit the safe use of this system in living systems. Therefore, further research to activate DNAzymes, e.g., by stable cavitation using auxiliaries like microbubbles^[^ ^38^, ^39^ ^]^ will be necessary. Moreover, the reduction of tissue damage through cavitation effects mandates short sonication times. Therefore, future work must address this by increasing the mechanochemical efficiency of the system even further. We expect that the amount of mismatches with the RCA product will be an important parameter to achieve a low activation barrier and short sonication times. Lastly, cellular uptake might also be a limiting factor, since typical DNA transfection methods involve the formation of DNA complexes with various cationic compounds, which then are taken up by the cells. In the RCA products, the DNA is present in a highly condensed form, which might lead to inefficient complexation and subsequently to low uptake, although cavitation effects might improve cell uptake through sonoporation.^[^ ^17^ ^]^ Once these challenges are addressed, we anticipate that the low cytotoxicity and high mechanochemical reactivity of DNA will enable its future use in biological systems.

## 4 Experimental Section

### Materials

All oligonucleotides were purchased from Biomers. The sequences are listed in Table 1, with the mismatches on the RCA template marked in bold. T4 DNA ligase and 10X ligation buffer were purchased from Thermo Fisher. Φ29 polymerase and 10X polymerase buffer were purchased from Biosearch Technologies. Tris and boric acid were purchased from Thermo Fisher. EDTA disodium dihydrate was purchased from Sigma. ROTI GelStain Red was purchased from Carl Roth.

### Padlock Ligation

The ligation was performed with a total reaction volume of 60 µL in 1X ligase buffer, containing 40 mm Tris‐HCl, 10 mm MgCl_2_, 10 mm DTT, and 0.5 mm ATP at pH 7.8. A template concentration of 40 µm and a primer concentration of 20 µm were used. The two strands were annealed by heating to 95 °C for 5 min, then cooling down by 1 °C every 2.5 min to 16 °C (standard annealing protocol). Afterwards, 10 U of T4 ligase was added and the mixture was incubated at 16 °C overnight. The ligation product was used without further purification. Gel electrophoresis was performed in TBE buffer (89 mM Tris, 89 mM boric acid, 2 mM EDTA, pH 8,0) and staining was performed with ROTI GelStain Red. Gel images were inverted using ImageJ and the contrast was enhanced to 2% saturated pixels.

### Rolling Circle Amplification

RCA was performed at a total reaction volume of 60 µL in 1X polymerase buffer, containing 50 mm Tris‐HCl, 10 mm (NH_4_)_2_SO_4_, 4 mm DTT, and 10 mm MgCl_2_ at pH 7.5. The ligation mixture (10 µL) was used. A total dNTP concentration of 15 mm was used, and the mixture of nucleotides was adjusted to the template sequence. The DNAzyme was added at a concentration of 26.7 µm, so the final concentration of 8 µm was reached at a volume of 200 µL. Finally, 30 U of Φ29 polymerase were added and the mixture was incubated at 30°C for 48 h. Gel electrophoresis was performed as described above.

### DNAzyme Activity Assays

All fluorescence measurements were performed with a SpectraMax M3 microplate reader by Molecular Devices. The wavelength settings were *λ*_exc_ = 495 nm and *λ*_em_ = 525 nm and the PMT gain was set to medium with 6 flashes per read. The DNAzyme activity assays were performed in a buffer containing 50 mm Tris, 6.25 mm CaCl_2_, and 2.75 mm MgCl_2_ at pH 7.9 with a total volume of 200 µL and a substrate concentration of 2 µm. For the deactivation experiments, the RCA/DNAzyme mixture was used without further purification.

### Release Experiments

Sonication experiments were performed under the same conditions as the activity assays, except the total volume was scaled up to 500 µL and the RCA reaction volume was scaled up to 150 µL. Sonication was performed with a Qsonica Q125 sonicator with a 3 mm probe (A12627PRB20). The frequency was 20 kHz with a pulse setting of 2 s on, 1 s off and the amplitude was set to 60%, resulting in a power density of 5.66 W cm^−2^ (2.89 MPa). The mixture was cooled with an ice bath, and the temperature was monitored with a FLIR TG165 thermal camera. After sonication, gel electrophoresis was performed as described above and the band intensity was determined using the gel analysis tool in ImageJ. The plots were normalized to their individual maximum. For the thermal release experiments, 200 µL solution was heated for 10 min to 50 or 100 °C, respectively. HIFU experiments were performed with a 0.66 MHz HIFU transducer by Precision Acoustics submerged in a water tank. The transducer was connected to a 33500B waveform generator by Keysight and an AG1021 amplifier by T&C Power Conversion. Furthermore, the HIFU setup was equipped with a DSOX3024T oscilloscope and a needle hydrophone by Precision Acoustics. 200 µL samples were prepared as described above in a Lumox 96 well plate with foil bottom by SARSTEDT and sealed with a 4titude moisture barrier seal. The bottom of the well plate was immersed in the water tank and sonicated for 10 min with a power density of 126.3 W∙cm^−2^ (1.94 MPa) at the focal point.

### Scanning Electron Microscopy

Samples (1–5 µL) were applied to a silicon wafer and dried at room temperature overnight. The wafer was attached to the sample holder using carbon tape and the sample was coated with ≈3 nm carbon using a Leica EM ACE600 sputter coater. Measurements were performed on a Hitachi SU9000 electron microscope using a secondary electron detector. An acceleration voltage of 30 keV at 10 µA was used. Particle diameters were measured using ImageJ.

### Statistical Analysis

All data in Figures 2, 4, and 5 is presented as mean ± SD, n = 3. Statistical significance in Figure 5 is determined using an unpaired two‐sample t‐test (*p* = 0.047), where significance was defined as *p* ≤ 0.05. Statistical analysis was performed using OriginPro 2018b.

## Conflict of Interest

The authors declare no conflict of interest.

## Supporting information

Supporting Information

## Data Availability

The data that support the findings of this study are openly available in [Zenodo] at [https://doi.org/10.5281/zenodo.8304891], reference number [40].
