# Supplementary material for: Mechanochemical Activation of DNAzyme by Ultrasound
Source: Adv Sci (Weinh). 2024 Feb 2;11(8):2306236. doi: 10.1002/advs.202306236 (PMC10885644; doi:10.1002/advs.202306236)
Supplement: Supplementary file 1 — Supporting Information [file ADVS-11-2306236-s001.pdf]

ADVANCED SCIENCE
Open Access

Supporting Information

for *Adv. Sci.*, DOI 10.1002/advs.202306236

Mechanochemical Activation of DNAzyme by Ultrasound

*Wolfgang H. Rath, Robert Göstl and Andreas Herrmann**

Supporting Information

Mechanochemical Activation of DNAzyme by Ultrasound

*Wolfgang H. Rath, Robert Göstl, and Andreas Herrmann**

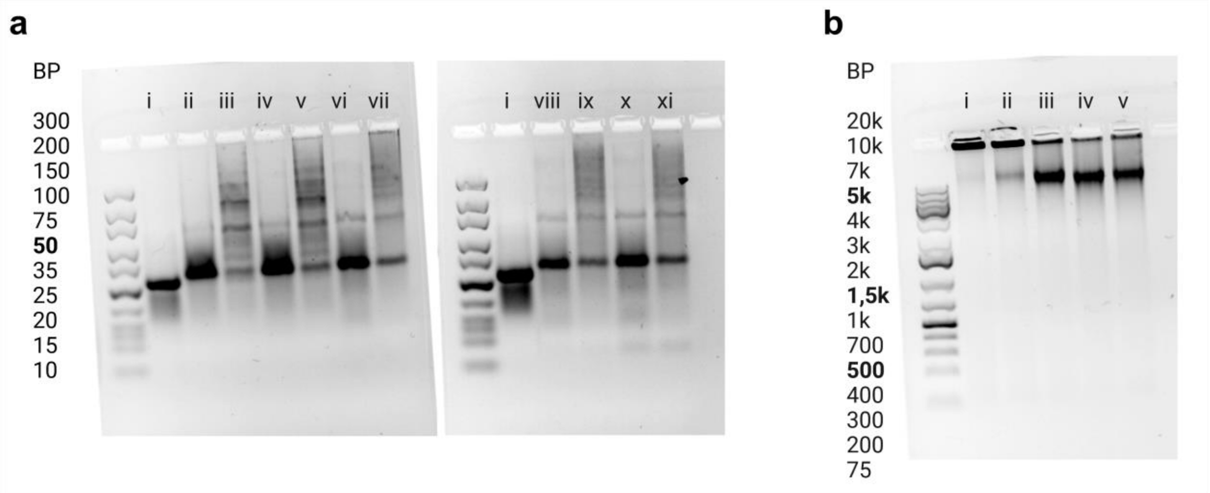

Figure S1. Synthesis of RCA products analyzed by agarose GE. (**a**) Padlock ligation, 4% agarose, ladder: GeneRuler ULR; lane i: linear template; ii: template:primer ratio 2:1, hybridized, no T4 ligase; iii: template:primer ratio 2:1, after ligation; iv and v: 1.5:1 ratio; vi and vii: 1:1 ratio; viii and ix: 1:1.5 ratio; x and xi: 1:2 ratio. (**b**) RCA reaction with different template:primer ratios, 0.8% agarose, ladder: GeneRuler 1 kb plus; lane i: 2:1 ratio, ii: 1.5:1 ratio, iii: 1:1 ratio, iv: 1:1.5 ratio, v: 1:2 ratio.

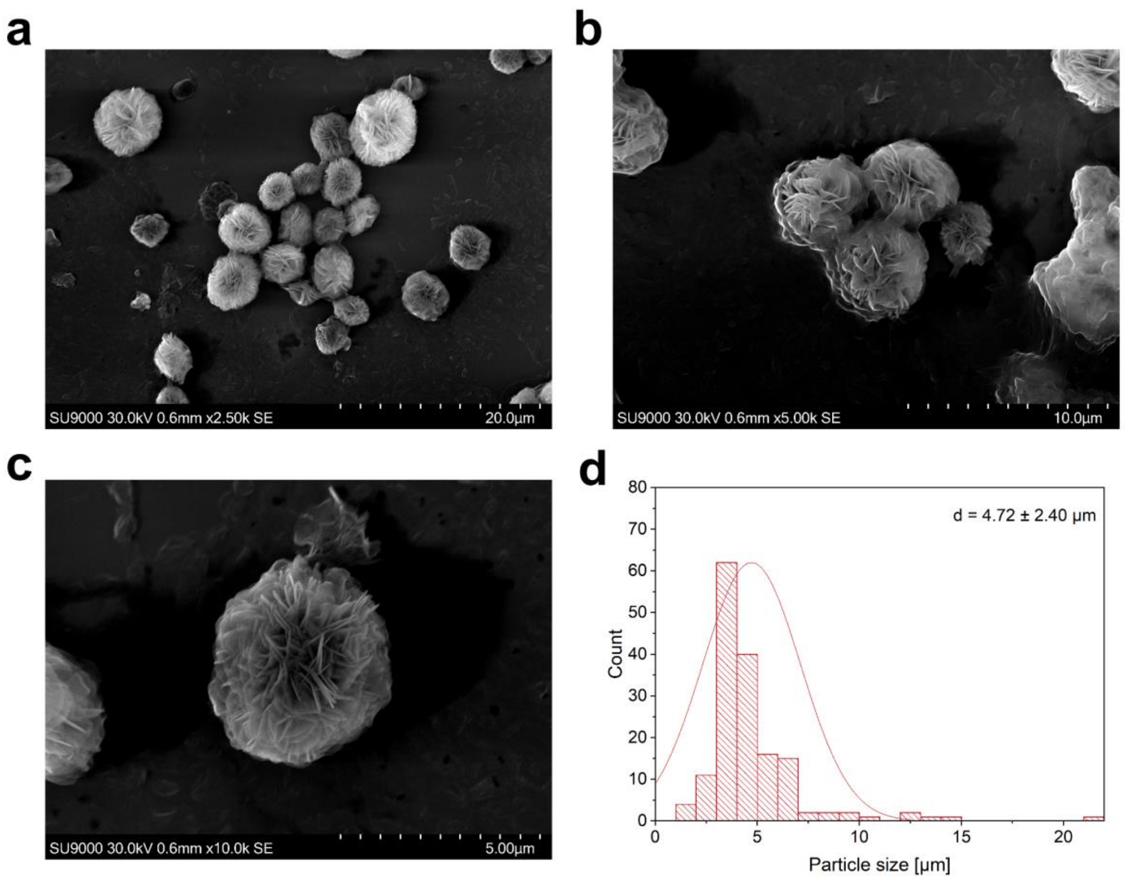

Figure S2. SEM images of DNFs before sonication. (**a**) 2500× magnification. (**b**) 5000× magnification. (**c**) 10000× magnification. (**d**) Particle size histogram. Mean ± SD from the mean. *N* = 160 particles.

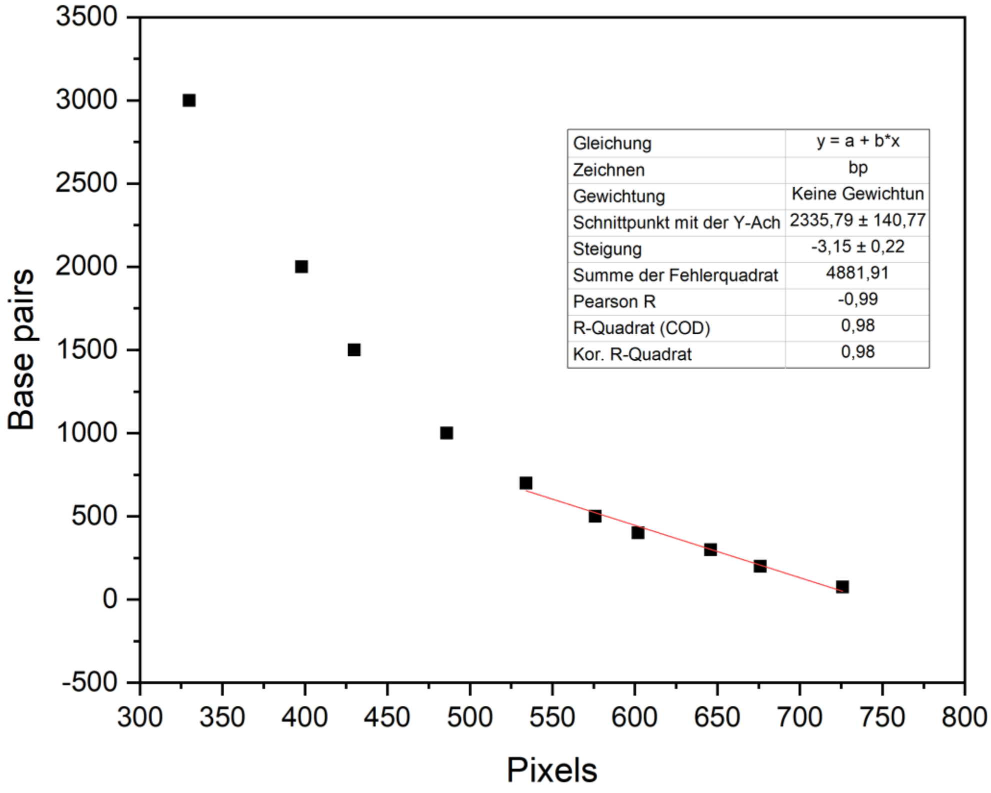

Figure S3. Calibration curve to determine DNA chain length. Maximum intensities after sonication were found at 647 bp (2 min), 338 bp (5 min), 301 bp (10 min), and 225 bp (20 min).

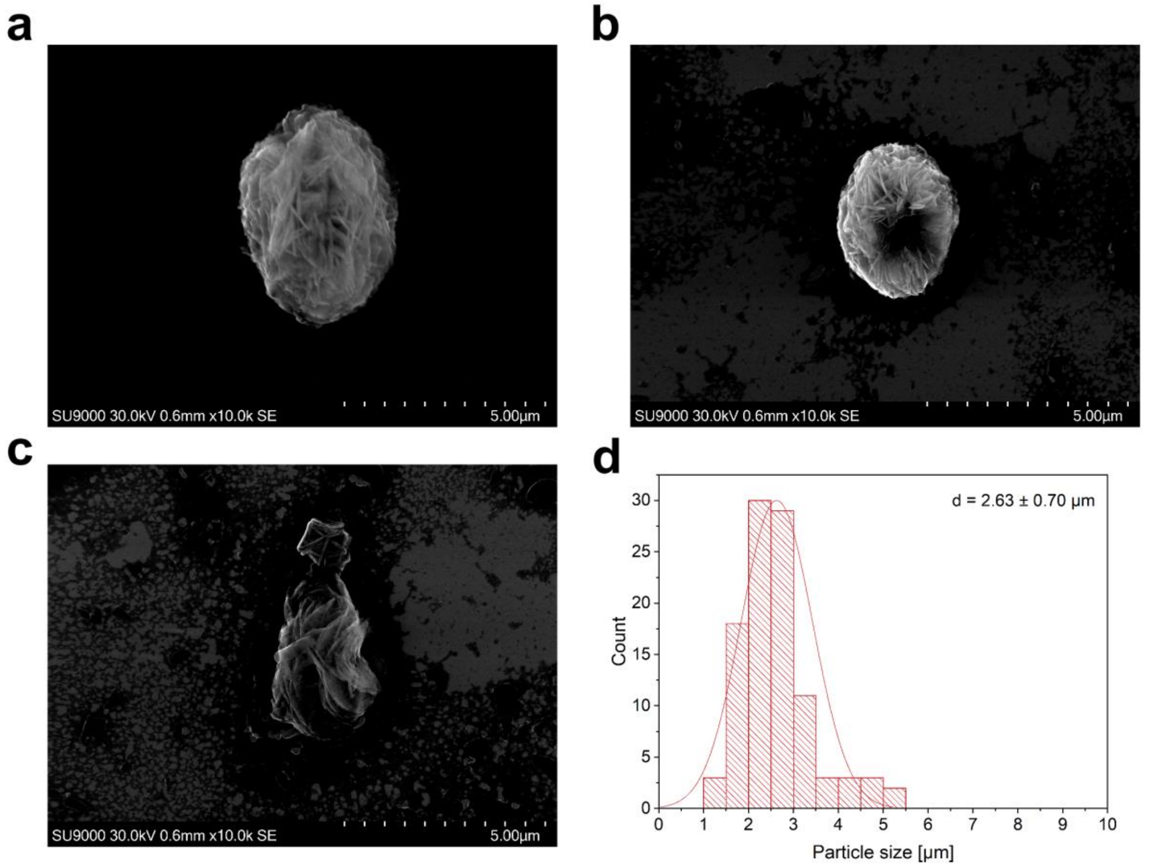

Figure S4. SEM micrographs of DNFs after sonication at f = 20 kHz. (**a-c**) 10000× magnification. (**d**) Particle size histogram. Mean ± SD from the mean. N = 100 particles.

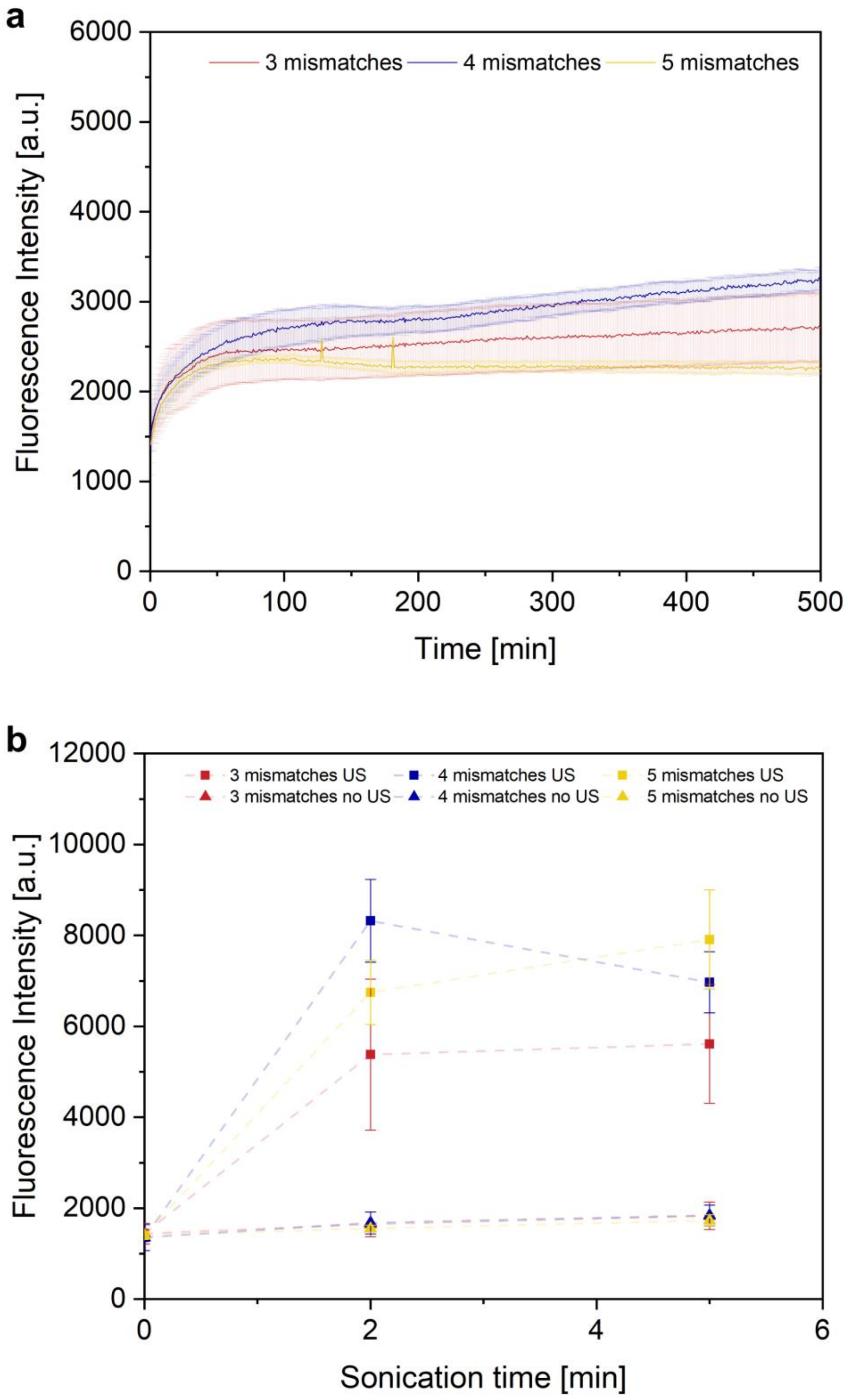

Figure S5. DNAzyme kinetics after loading onto RCA product with 3, 4, and 5 mismatches, 2 µM substrate, 8 µM DNAzyme, all data presented as mean ± SD from the mean, N = 3 experiments. (**a**) Deactivation of DNAzyme through hybridization to RCA products. (**b**) Maximum fluorescence observed after sonication with 20 kHz US.
